# Supplementary material for: Comparative multiomics analysis of cell physiological state after culture in a basket bioreactor
Source: Sci Rep. 2022 Nov 23;12:20161. doi: 10.1038/s41598-022-24687-4 (PMC9686226; doi:10.1038/s41598-022-24687-4)
Supplement: Supplementary file 1 — Supplementary Information 1. [file 41598_2022_24687_MOESM1_ESM.zip › raw data/Transcriptomic raw data/6.Enrichment/1.GO/TE1vsTC1/ALL/TE1vsTC1.all_GObar.pdf]

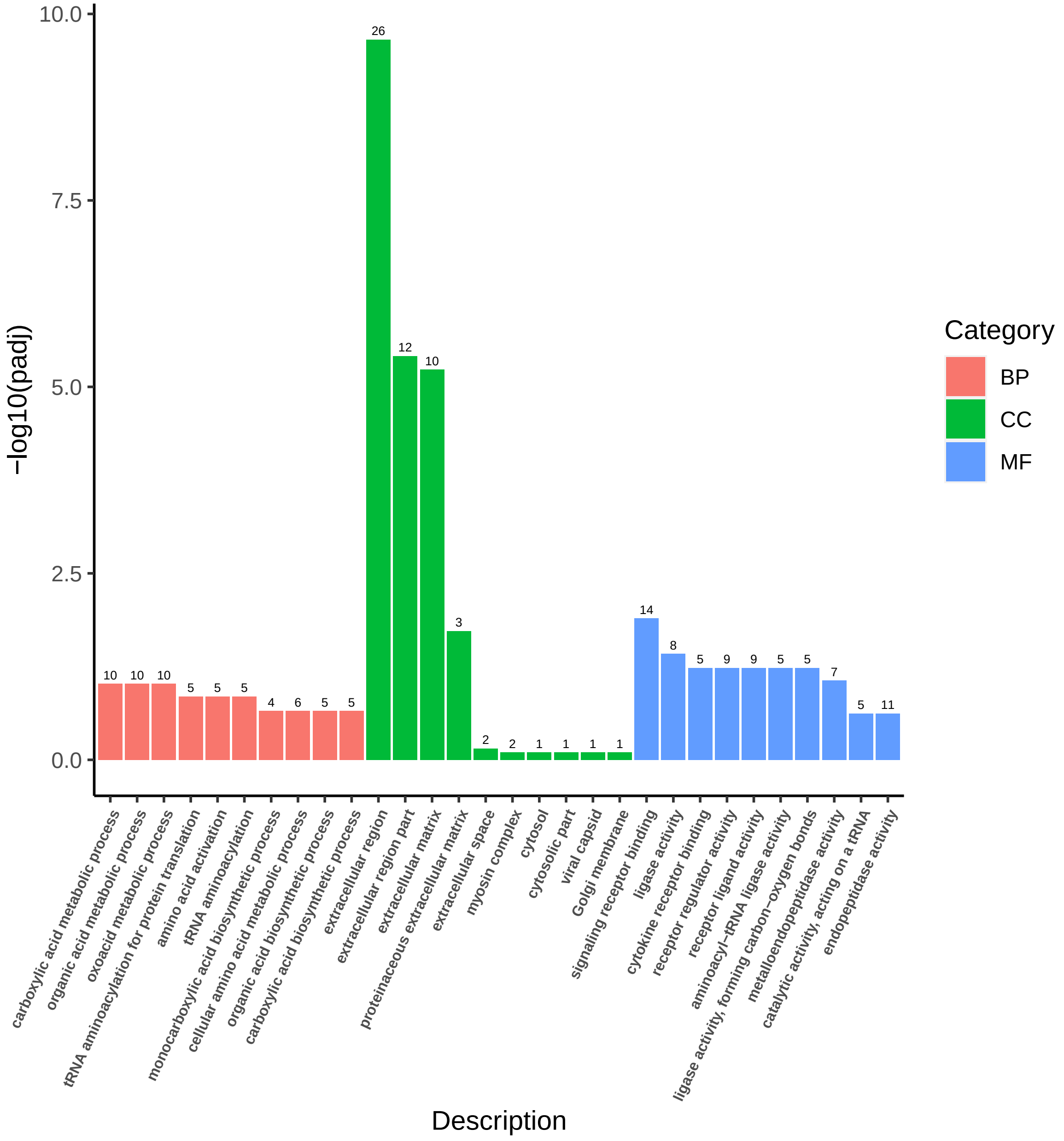

10.0
7.5
5.0
2.5
0.0
−log10(padj)
26
12
10
3
2
2
1
1
1
1
10
10
10
5
5
5
4
6
5
5
14
8
5
9
9
5
5
7
5
11
carboxylic acid metabolic process
organic acid metabolic process
oxoacid metabolic process
tRNA aminoacylation for protein translation
amino acid activation
tRNA aminoacylation
monocarboxylic acid biosynthetic process
cellular amino acid metabolic process
organic acid biosynthetic process
carboxylic acid biosynthetic process
extracellular region
extracellular region part
extracellular matrix
proteinaceous extracellular matrix
extracellular space
myosin complex
cytosol
cytosolic part
viral capsid
Golgi membrane
signaling receptor binding
ligase activity
cytokine receptor binding
receptor regulator activity
receptor ligand activity
aminoacyl−tRNA ligase activity
ligase activity, forming carbon−oxygen bonds
metalloendopeptidase activity
catalytic activity, acting on a tRNA
endopeptidase activity
Description
Category
BP
CC
MF
